# Microscopic Study of the Spinodal Decomposition of Supported Eutectic Droplets During Cooling: PtGe/Ge{110}

**DOI:** 10.1021/acs.jpcc.2c01356

**Published:** 2022-06-30

**Authors:** Zhiguo Zhang, Bene Poelsema, Harold J.W. Zandvliet, Arie van Houselt

**Affiliations:** Physics of Interfaces and Nanomaterials, MESA^+^ Institute for Nanotechnology, University of Twente, P.O. Box 217, Enschede 7500AE, The Netherlands

## Abstract

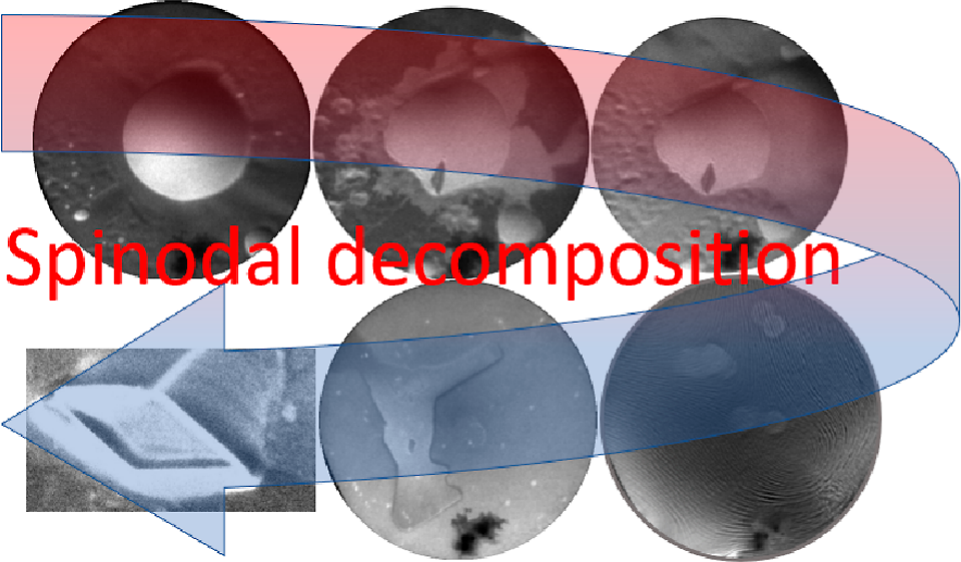

We embarked on an *in situ* low-energy electron microscopy, photo-electron emission microscopy, and selected area low-energy electron diffraction study during the cooling of huge eutectic droplets through the critical stages of the eutectic transition. On this journey through uncharted waters, we revealed an expected initial shrinking of the exposed area of the droplet, followed by an unanticipated expansion. We attribute this behavior to an initial fast amorphization of the interface between the droplet and surface, followed by the recrystallization of Ge expelled from the droplet at the interface. As a major surprise, we discovered the emergence of extensive “spaghetti”-like patterns, which are rationalized in terms of parallel Ge ripples oriented along, mainly, [−554] and [−55–4] directions. They emerge during spinodal decomposition when passing the eutectic temperature of the system. Their sides are defined by Ge{111} and Ge{11–1} vicinals covered with Pt-modified (√3 × √3) superstructures. The distance between adjacent ripples is about 18 nm.

## Introduction

The emergence of eutectic droplets on solid surfaces and their temporal evolution above the eutectic temperature have received proper attention.^1−11^ It has been well documented that, under the influence of a temperature gradient, eutectic droplets are thermodynamically driven toward the hottest available location at the surface and grow through, mainly, Smoluchowski ripening, that is, coalescence and mergence. This leads in the ideal case to one big droplet at the thermal summit and in practice to the accumulation of few large droplets close to the center. We have recently communicated a compelling example for PtGe on Ge{110}.^12,13^ While *in situ* information on the droplet dynamics above the eutectic temperature is readily accessible, there are no reports about the evolution during cooling through the eutectic transition. Only postmortem analysis is employed to show, for instance, by TEM that crystalline AB-remainders of the eutectic droplets reside on the pedestals of B precipitated from the droplet during cooling on substrate B.^3−5,8^ However, *in situ* information on the crystallization of eutectic droplets at the very moment when the system is driven through the eutectic transition during cooling is still lacking. It appears that our current knowledge of the system PtGe/Ge{110} offers a promising opportunity to gain *in situ* more insights into the processes that are active during the spinodal decomposition of eutectic droplets. We start with a microscopic view of a relatively small area around the hottest spot at the surface and thus with a large droplet and consequently a high local Pt concentration. During cooling down, the position of the hottest spot hardly changes, and as a result, the thermal gradient-induced motion of droplets is minimal. This is of importance as the experiment can be executed only once after depositing Pt on the virgin Ge(110) surface. Further away from the hottest spot, the local Pt concentration is lower after a prolonged experiment. This allows the experimental evaluation for both high and local Pt concentrations on a single substrate under the same experimental conditions.

The mirror side of Ge evolution is the coincident emergence of a large, compact, Ge_2_Pt{001} rhombic crystallite, located immediately next to the center and aligned with its *a*-axis under an angle of 20° wrt the Ge{110}-[001] direction. Further off-center (a few millimeters), extended orthorhombic Ge_2_Pt{101} crystals emerge, which are aligned with Ge{110} with its Ge_2_Pt *b*-axis parallel to Ge[001] at an almost perfect lattice match. These thin islands are stabilized by quantum size effects.

In this study, we apply PEEM (photo-electron emission microscopy), LEEM (low-energy-electron-microscopy), and μLEED (selected area low-energy electron diffraction) to monitor the structural changes *in situ* during cooling through the eutectic temperature. Upon cooling toward the eutectic temperature *t*, we find an unanticipated “breathing” of the wetting angle and, consequently, of the exposed area of the droplet, which we trace back to the structural changes at the droplet–substrate interface. In addition, we observe the emergence of “spaghetti”-like structures upon cooling below the eutectic temperature, which are rationalized in terms of a rippled spreading layer of pure Ge around the original droplet. In this process, a crucial role is taken by a Pt-containing (3 × 3) wetting layer on vicinal (111) Ge facets that constitute the ripples developing at the Ge{110} surface. Furthermore, at different surface locations (and thus different local Pt concentrations), differently oriented Ge_2_Pt crystallites are identified.

## Experimental Section

The experiments have been conducted with an ELMITEC LEEM-III instrument with a base pressure of 10^–10^ mbar. In PEEM, the surface was illuminated with a 100 W mercury discharge lamp (λ = 0.253 μm) incident at 16° from the surface plane. The absolute temperature reading is estimated to be correct within ca. 25 K and calibrated by making the reasonable assumption that the eutectic temperature at the surface equals that of the bulk (1050 K for GePt). A Ge{110} substrate, 10 × 10 mm^2^, nominally flat, single-side-polished, n-type Ge(110) crystal (MTI Corporation, *R* > 50 Ω cm), has been degassed for about 24 h at 700 K, followed by several cycles of argon ion bombardment and flash annealing by e-beam bombardment at a temperature exceeding 1000 K. Subsequently, Pt is deposited from a resistively heated W wire wrapped with high-purity (99.995%) Pt (Alfa Aesar). The structure of the clean surface has been examined using LEED. Like the findings in ref (14), we also observed a c(8 × 10) structure at lower substrate temperatures, while above 1050 K, only the nonreconstructed (1 × 1) structure is observed.

## Results and Discussion

**Figure 1:**
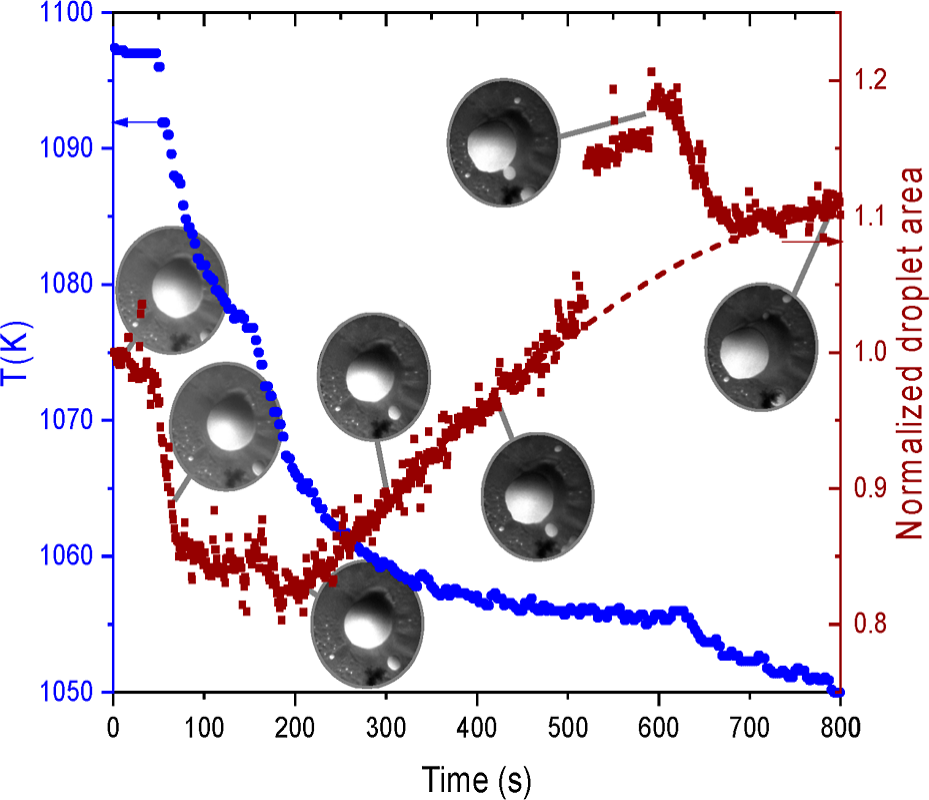
Seven snapshots (points of time indicated by gray connectors) from a PEEM movie [field of view (FoV), 150 μm] taken during cooling toward the eutectic temperature of 1050 K taken at strategic temperatures. The temperature is given on the left-hand scale. The projected area of the large eutectic droplet, normalized to its starting value, is given on the right-hand scale. The transient increase around 500–650 s is due to the impossibility to separate the area from that of the merging small cluster from below. The relevant timescale is plotted along the abscissa.

At the start of the current experiment, the surface was prepared as described above, followed by a prolonged period (>8 h), at a temperature of about 50 K above the eutectic temperature. As a result, a large eutectic cluster is situated in the center at the hottest location at the surface, and several smaller ones are still on their way to this center. We follow the lifeline of this object during a gentle cool-down. Initially, as can be observed in the accompanying PEEM movie,^15^ the cluster moves a little due to a slight change of the temperature profile: it remains at the local hot spot, just microns away from its starting position. A few characteristic snapshots of the movie are reproduced in Figure 1. We use spherical caps as a good approximation of the eutectic droplet with, initially, a flat interface with the Ge{110} substrate. Quite minor deviations from circular geometry are attributed to the step architecture, such as step-bunches.^13^ The radius of curvature of the spherical cap of the major eutectic droplet (bright objects) amounts to *R*_C_ = 90 μm, and the wetting angle θ_w_ equals 20°.^12^ The volume of the spherical cap is given by
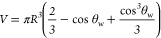
1

For the numbers above, we obtain *V* = 8.2 × 10^3^ μm^3^. Or, with the atomic volume of 1 Ge atom per 22.6 × 10^–12^ μm^3^ (bulk Ge), we find 3.6 × 10^14^ atoms inside the spherical droplet, assuming that the atomic volumes of Pt and Ge are identical in this crude estimate.

**Figure 2:**
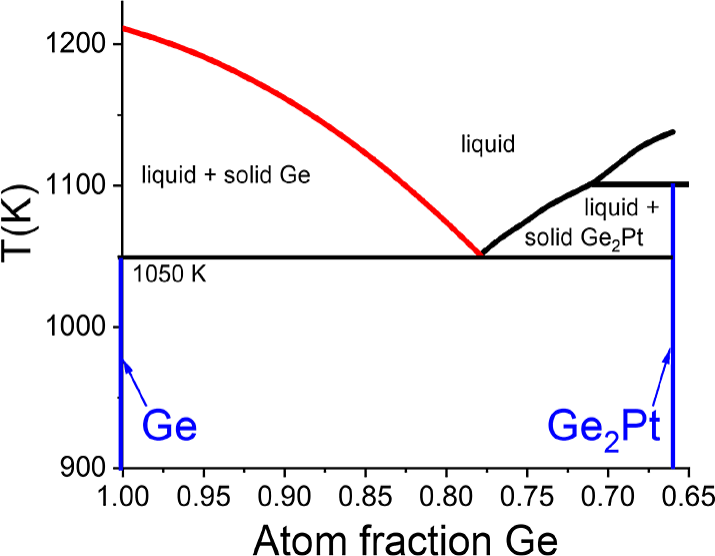
Bulk phase diagram of Ge–Pt. Data replotted from ref (17).

As illustrated by Figure 1, immediately upon cooling down, the exposed area of the eutectic droplet decreases. It decreases by about 18% upon a temperature drop of 19 K. Qualitatively, this behavior confirms the expectation as derived from the Ge–Pt bulk phase diagram in Figure 2. In equilibrium, the system moves during cooling along the liquidus line marked in red toward the eutectic point. As the number of Pt atoms inside the droplet remains constant and the relative content of Ge decreases, the segregating Ge atoms are incorporated into/onto the Ge substrate at the droplet–substrate interface. For completeness, we remark that Ge_2_Pt crystallites also emerge below the eutectic temperature,^16^ which will be discussed in more detail at the end of the paper. Immediately upon cooling, one observes a decrease of the projected area of the droplet, as illustrated in Figure 1. This corresponds qualitatively to the expected decrease of the volume, provided the wetting angle remains constant. However, we detect a substantial quantitative problem: we measure a decrease of the projected area of about 18% during a temperature drop of 19 K, while from the slope of the liquidus line in the considered temperature range, a volume drop by ca. 11% is expected, resulting into a decrease of the projected area of only 7%. In other words, the loss of Ge atoms inside the droplet is insufficient to account for the observed decay of the exposed area of the eutectic droplet. This can be rationalized in terms of a change of the wetting angle. As is well known, the wetting angle is given by Young’s equation
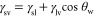
2with γ_sv_, γ_sl_, and γ_lv_ being the tension of, respectively, the substrate–vapor, the substrate–liquid, and the liquid–vapor interfaces, while θ_w_ denotes the wetting angle. Naively, one would expect that within the small variations of the Ge content inside the droplet only marginal changes in the interfacial tensions occur and thus the wetting angle would stay constant. However, the sedimentation of Ge at the droplet–Ge{110} interface during the cooling of the eutectic may well result in an increasing kinetic roughness at this interface. This roughness would, according to Wenzel,^18^ lead to a reduction of the wetting angle
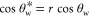
3with θ_w_* the wetting angle on the rough surface and *r* the roughness defined as the real surface divided by the nominal surface, and thus by definition, *r* > 1. Consequently, this would result in an increase of the projected area. Therefore, kinetic roughening must be excluded as the course for the discrepancy we ran into. We do realize that an increase of γ_sl_ would lead to an increased wetting angle and potentially would lift the apparent discrepancy. To advance along this route, we consider how much material is segregated from the spherical droplet segment to the droplet–substrate interface.

The base area of our spherical droplet segment with the radius of curvature *R*_C_ = 90 μm and wetting angle θ_w_ = 20° amounts to ∼3000 μm^2^. One atom in the Ge(110) surface covers 1.13 × 10^–7^ μm^2^; in other words, the circular base plane of the droplet counts roughly 2.6 × 10^10^ atoms. The deposition of all Ge atoms in the droplet at the base would therefore result in a growth of 1.4 × 10^4^ Ge(110) monolayers. During the 19 K temperature decrease in about 25 s, in line with the quick drop in exposed area exhibited in Figure 1, the Ge content of the droplet sinks from 0.85 to 0.831, and therefore 270 monolayers (ML) are deposited at the interface at an estimated rate of 11 ML/s. This is too fast to secure the crystalline growth, especially for semiconductors, and amorphous Ge will grow at the interface (in line with Figure 3b in the Supporting Information of ref (19)). At the amorphous interface, the density of broken bonds is high and, therefore, the corresponding interface tension γ_sl_ will be relatively high. Accordingly, we attribute the unanticipated shrinking of the exposed area of the cooling down eutectic droplet to the kinetic amorphization of the growth front at the droplet–substrate interface.

This scenario offers a natural framework for understanding the observed evolution of the projected area of the eutectic during later stages, as illustrated in Figure 1. A lower cooling rate gives rise to recrystallization of the amorphous crystalline interface. The liquid–substrate interface tension will thus decrease, and the wetting angle decreases accordingly. Further slow decrease of the cooling rate then leads to lower wetting angles according to Wenzel (eq 3), and the final exposed area even overwhelms the initial one.

The behavior of the exposed area as a function of decreasing temperature clearly reveals that the two active processes (1) kinetic amorphization and (2) crystallization at the droplet occur not consecutively but simultaneously. These processes compete with amorphization dominance during fast temperature decay and crystallization dominance at slow temperature decay rates. In this case, the temperature adjustment was controlled in three steps, and each time an initial drop is followed, after some time, by an increasing tendency of the exposed area when the temperature decay rate decreases. This behavior is in line with the scenario outlined above.

When passing through the eutectic temperature, two events occur simultaneously: (1) a spreading of the material originating from the Ge–Pt cluster and (2) a partial crystallization of the former droplet remainders. In terms of the expected spinodal decomposition, one would naively conclude that the spreading results mainly from Ge incorporation in the Ge{110} substrate, and the crystallization at the position of the former droplet would result in PtGe_2_ crystallites. This appears to be confirmed, however, with an unanticipated twist, as we will discuss in detail further below. In this evaluation scheme, our exemplary spherically capped droplet with *R*_C_ = 90 μm and θ_w_ = 20°, when cooled down from 1100 K through the critical temperature, contains 7.4 × 10^13^ Ge atoms which need to be reincorporated into the Ge{110} substrate. If these are equally spread over the area of FoV, of 150 μm in Figure 1, it would imply a deposition of 470 ML and a corresponding height increase of slightly less than 0.1 μm. Anyway, one is bound to observe major mass transport near the center of the sample during spinodal decomposition. This is in line with the measured diffusion rates for the related system Si/Ge{110}.^20^

**Figure 3:**
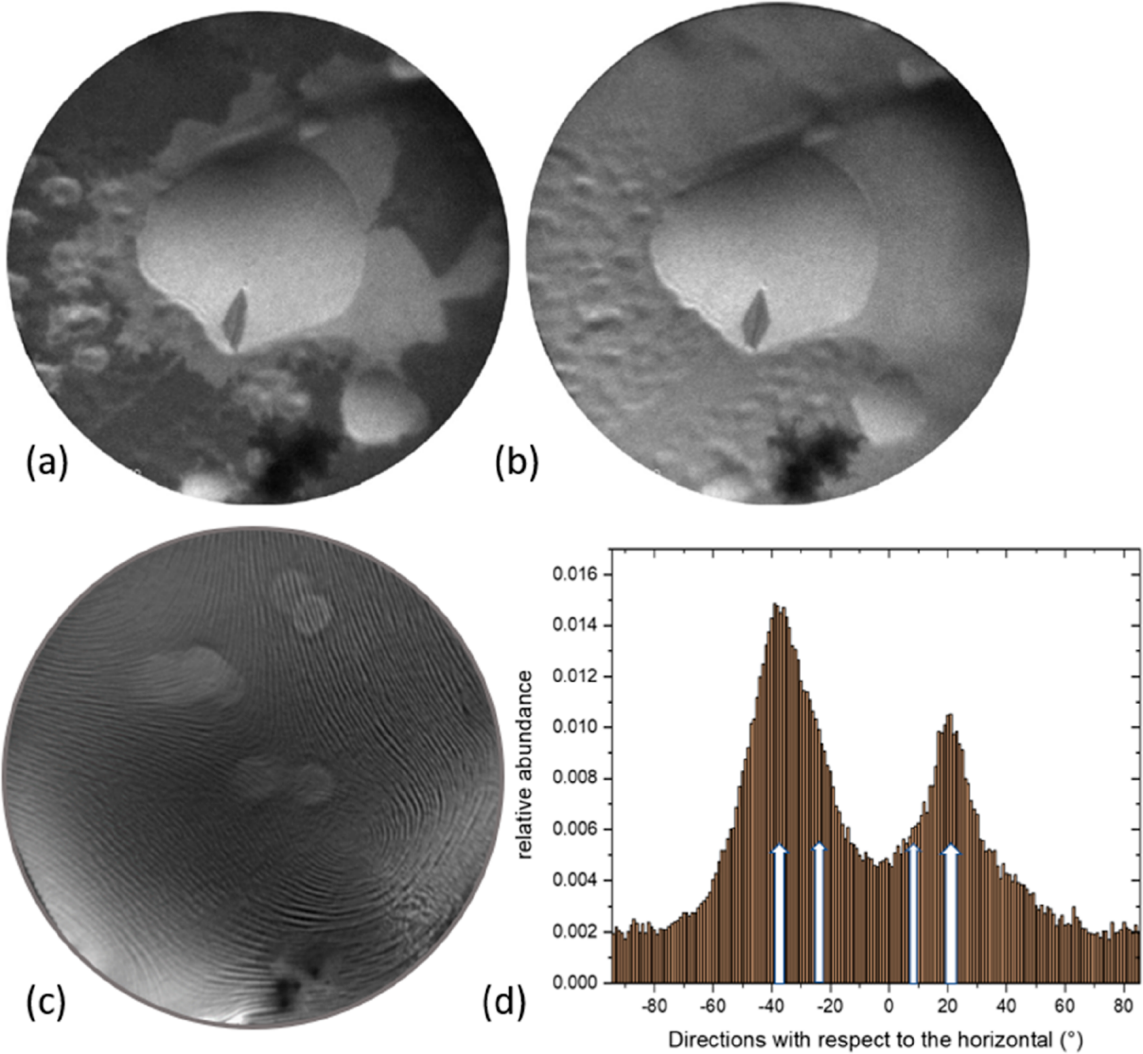
Two snapshots [(a) 990 K] and [(b) 970 K] taken during cooling through the eutectic transition. (c) Mirror image (start voltage—0.8 V) taken at room temperature of the spreading film (see text), with a FoV of 20 μm. At the somewhat brighter circular areas, the electron beam dwelled for a longer term. (d) Histogram of the local directions of the “spaghettis” in (a) gathered in 1° wide bins. The numbers integrate to unity. The white arrows are discussed in the text further below.

**Figure 4:**
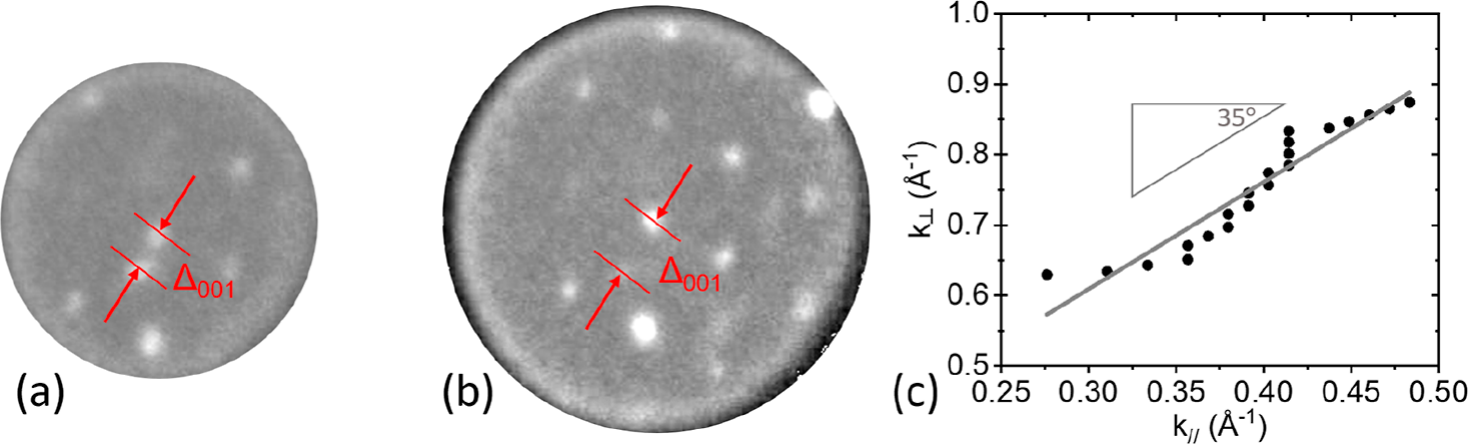
μLEED patterns taken at 1.9 eV (a) and 3.3 eV (b), effective aperture size of 1.4 μm, room temperature. The arrows indicate the (0, 0) spots of the (110) substrate structure and that of the (distorted) hexagonal structure in the image. The distance between these spots along [001] is Δ_001_. Note that the circles represent the Ewald sphere which scales with *E*^1/2^. (c) Normal component of the wave vector (ordinate) versus Δ_001_ (abscissa) of the diffracted electrons.

A first impression of the events is provided by the snapshots in Figure 3a,b. The PEEM data shows that a film spreads from the congealing droplet, and simultaneously the original droplet appears to solidify as well. First, we concentrate on the spreading film, which soon covers the entire FoV. After further cooling down of the film, one obtains evidence for remarkable structures that emerge during the solidification of the spreading layer. An example is shown by the room-temperature mirror image (−0.8 V) LEEM picture in Figure 3c, taken from the spreading layer at room temperature. A highly surprising and intriguing pattern has evolved, which we will refer to as “spaghetti” below. Across the FoV of 20 μm, the spaghetti pattern appears quite homogeneous. It is emphasized that the mirror image reveals work function variations which could be related to morphology (likely) and/or chemical composition (less likely). We find an obvious preference for the periodicity normal to the strings, which amounts to about 18 nm. The brighter circular areas result from a relatively long-term interrogation of the structures by applying μLEED using the smallest available aperture of 1.4 μm. The contrast change is the result of a slight electron beam-induced change of the local work function, but the figure clearly documents that there is no influence on the morphology. At first sight, the directionality of the spaghettis is quite random, but a closer look reveals the strong preference for two azimuth directions, as shown by the directional histogram in Figure 3d. These preferred directions are about 60° apart. To gain a deeper insight into the complex rearrangement events at the surface during spinodal decomposition, we apply μLEED on the “spaghettis” in a carefully selected area (the bright areas in Figure 3c) using an aperture of 1.4 μm. Figure 4a,b exhibits measured diffraction patterns at electron energies of 1.9 and 3.3 eV. One distinguishes a distorted hexagonal pattern and an additional peak (indicated by the lower arrow), which is attributed to the specular peak of the (110) substrate (or areas parallel to this).

Upon increasing the electron energy, the distance between this peak and the specular spot of the distorted hexagonal pattern (higher arrow), referred to as Δ_001_, becomes larger. The value of the normal component of the wave vector of the diffracted electrons versus the parallel component change along [001] is plotted in Figure 4c. The relative motion of diffraction spots reveals the presence of facets at the surface (see for a detailed review refs (21) and (22)). From a plot of the vertical component of the wave vector change versus its parallel component for several electron energies, one may derive the angle between different facets. Such a plot is made available in Figure 4c, and we extract 35°. For a cubic crystal, the angle between (111) and (110) planes amounts to 35.26°, and we can safely conclude that we deal with the emergence of (111) facets on the (110) substrate. This is further reinforced by the fact that (111) facets are quite stable and are often the constituents of reconstructed (110) surfaces, both for metals and for semiconductor surfaces.^23−26^ Also, the presence of a (distorted) hexagonal structure in Figure 4a,b hints into this direction. The intensity of the specular (110) spot decreases strongly with increasing electron energy. This feature is attributed to the fact that the transfer width of the instrument is a strong function of the electron energy, and it gets only exceptionally large at zero energy.^27^ The disappearance of the (110) specular spot already at low energies reveals that, overall, the (111) facets dominate over (110) areas.

**Figure 5:**
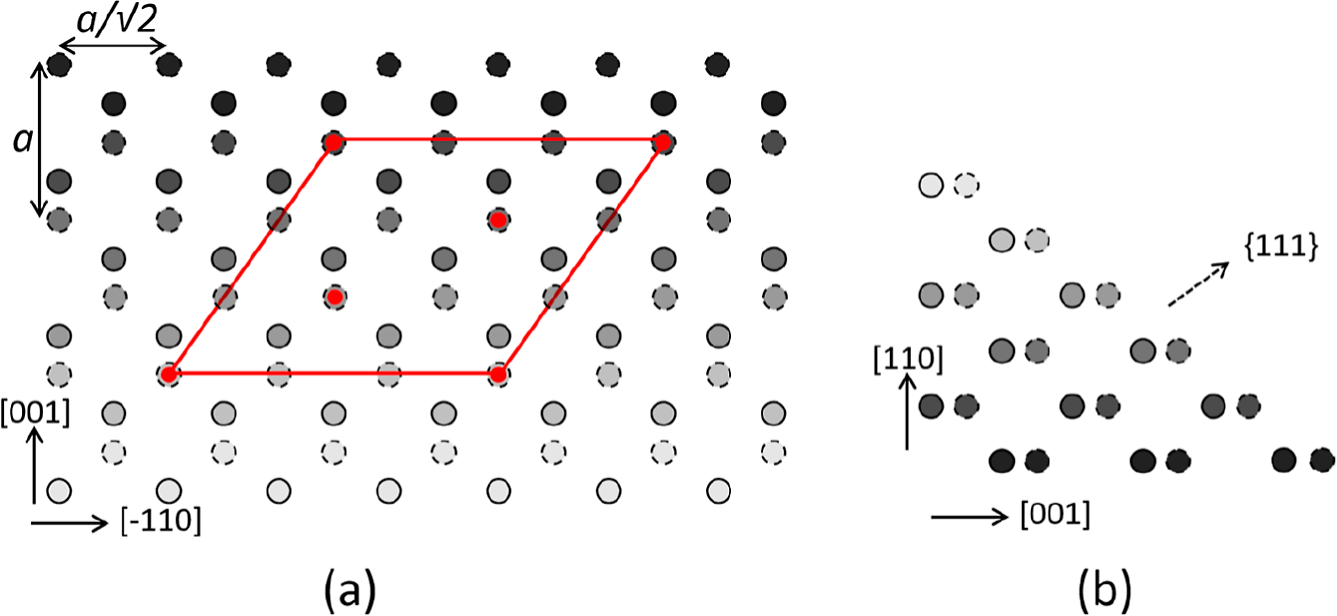
(a) Top view of the Ge{110} surface with a (111) facet. The edge runs along the [−110] direction (*x*-axis), and the *y*-axis corresponds to the [001] direction. The atoms with different outlines belong to different sublattices of the diamond structure. The darker appearances denote atoms at consecutively lower (110) levels. See text for the significance of the red dots. (b) Side view, with edges running along the [001] direction (*x*-axis) and the [110] direction (*y*-axis).

Figure 5 shows a sketch of the Ge{110} surface with a (111) facet. Figure 5a shows a top view of a projection on the (110) surface, while Figure 5b shows a side view. The atoms indicated by open and closed circles belong to different sublattices of the diamond structure. In Figure 5a, we see the outermost [−110] strings of atoms at different (110) levels. The circles with stepwise-increasing gray values denote Ge atoms at consecutively lower levels.

**Figure 6:**
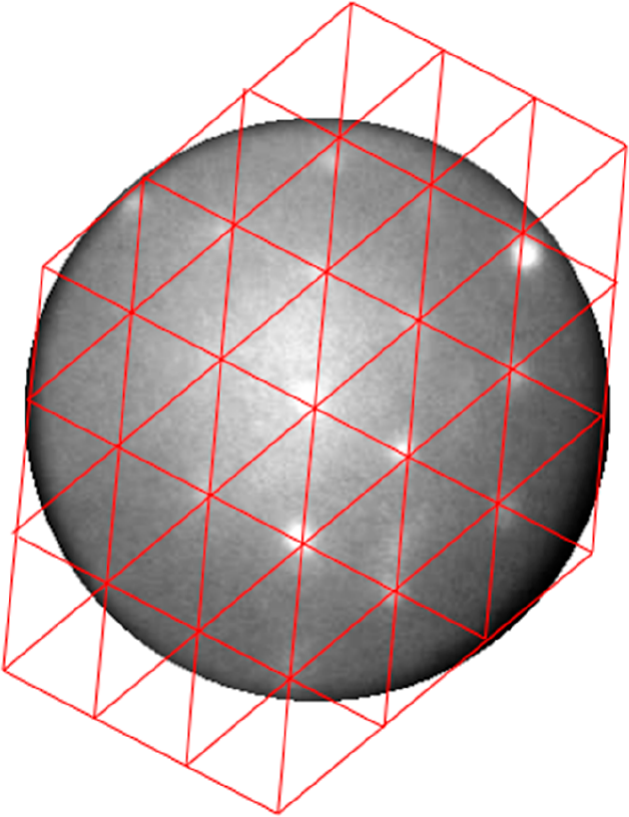
Measured diffraction pattern at 4.1 eV and room temperature. The red grid is elongated along the [11–2] direction (real space indication) by a factor of 1/cos(35°).

Note that in this (110) projection the distance between the successive [−110] atom strings is smaller by a factor, cos(35.26) = 0.82, than their distance within the (111) facet. This geometric fact causes the abovementioned distortion of the diffraction pattern of superstructures at the facets. A decrease in real space gives rise to an elongation in reciprocal space. This is exactly what we observe, as illustrated in the diffraction pattern taken at 4.1 eV and shown in Figure 6. We have elongated an ideal hexagonal raster along the real space [11–2] direction by a factor cos^–1^(35) (red grid) and find, neglecting minor residual image distortions, an almost perfect mapping of the measured diffraction peaks. This result is a confirmation for the already concluded presence of (111) vicinals.

The diffraction pattern reveals a (3 × 3) reconstructed hexagonal pattern. This is attributed to a Pt-containing cover layer on the (111) facets. We suggest that one-third of the Ge atoms in the topmost layer of one of the sublattices is replaced by Pt. These are indicated by the red dots in Figure 5. We emphasize that the surface tensions for clean FCC (111), (100), and (110) surfaces increase in this sequence. Sometimes, the energy gain of (111) facets, when compared to the (110) termination, may even outweigh the unfavorable correspondingly larger surface area, leading to a (2 × 1) reconstruction of the clean surface.^24,25^ The presence of a metal-induced reconstruction of (111)-type may well influence the subtle energy balance in favor of the formation of (111) facets. This may even lead to a preference of reconstructed (111) facets above (100) areas. An example of the latter is the Au-induced giant missing row reconstruction of Ge{001} with (√3 × √3) reconstructed (111) facets.^26^ The current observations with Pt-induced (3 × 3) structures on large (111) facets reveal a similar mechanism. We suggest that one-third of the Ge atoms in the topmost layer of one of the Ge sublattices is replaced by Pt, as indicated by the red dots in Figure 5a, resulting in a (3 × 3) structure. Note that the edge in Figure 5a runs along the [−110] direction. Before we move on to a more detailed contemplation, we first want to note that the location of the incorporated Pt atoms at the corners and the long diagonal gives rise to three Pt atoms in the (3 × 3) structure, and a simpler identification of the vital unit cell is a (√3 × √3) one, with one Pt and two Ge atoms in the unit cell. We prefer to work with this basic building block from now onward.

**Figure 7:**
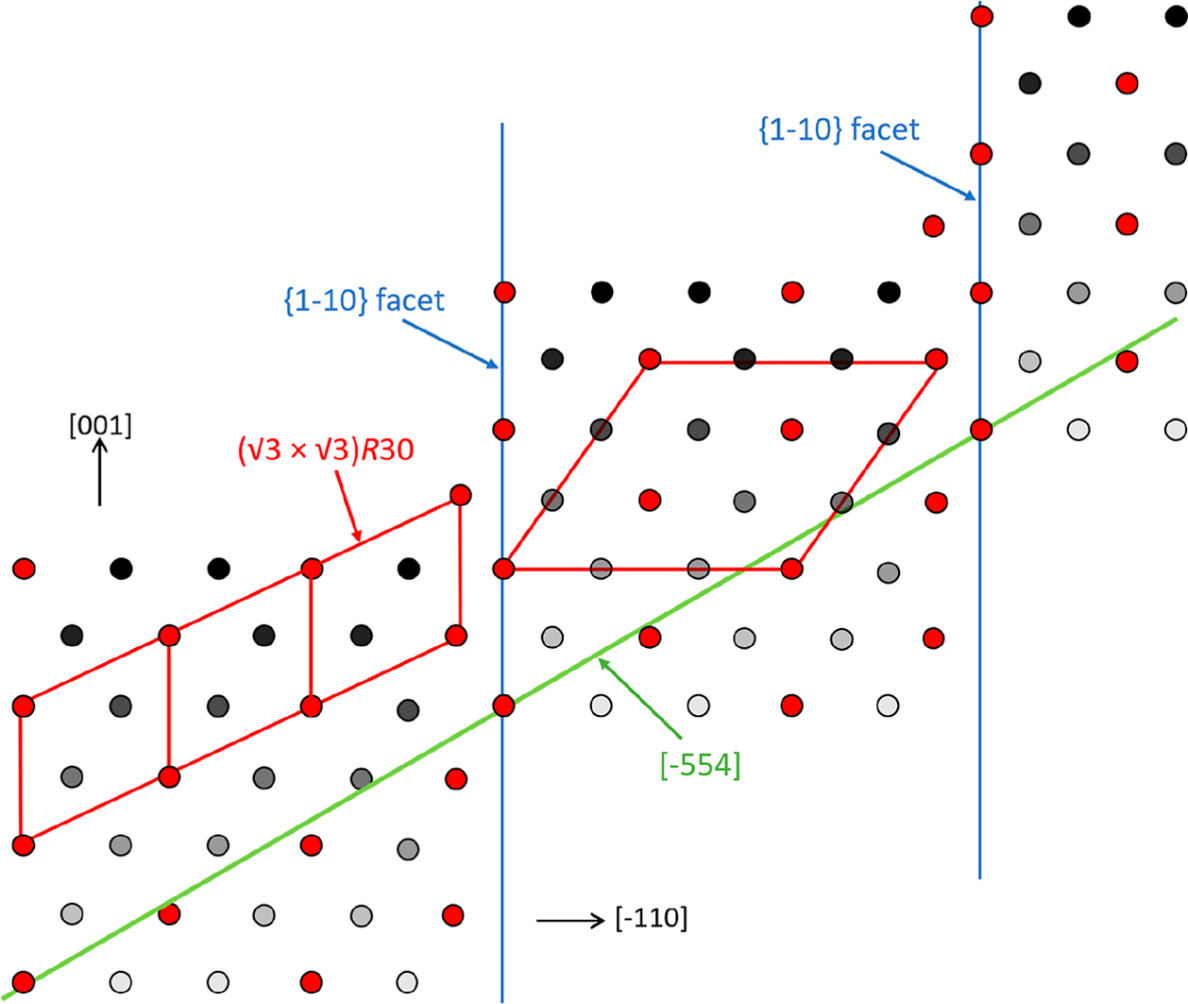
Top view of a Ge ripple oriented roughly along [−211] on the Ge{110} surface. A {111} facet is shown with increasingly darker atoms at consecutively lower lying lattice positions. Pt atoms that have exchanged positions with Ge are colored red. The blue lines indicate atomic (multi) steps on the {111} facet. The blue line is the intersection of the {110} and {111} surface planes. The solid red grids at the left-hand side show (√3 × √3) unit cells on {111}. A full (3 × 3) cell in {110} projection is indicated by the larger red parallelogram on the central terrace. For simplicity, only one of the two sublattices is shown. Atoms of both sublattices form a bilayer on (111)-oriented facets. The uppermost atoms of the bilayer on {111} and {11–1} layers originate from different sublattices. The intersection of {735}, that is, the stepped {111} face (see text), with {110} is indicated by the green line along [−554]. Note that for symmetry reasons an equivalent ripple occurs along [−55–4].

It appears attractive to use the simple and straightforward model depicted in Figure 5 as the explanation for the formation of Ge ripples, oriented along [−110], with Pt-modified and stabilized {111} facets and mirrored {11–1} facets on the opposite side. However, such a strong preference for the unilateral orientation of the ripples along [−110] is at variance with the observation displayed in Figure 3d, which clearly reveals a preference for *two* equivalent azimuthal directions which are about 60° apart. The reason for this at first sight unexpected result must be searched for in the strong anisotropy of the (√3 × √3) structure which is responsible for the evolution of the ripples in the first place. We suggest that the ripples are aligned along the directions most densely packed with Pt atoms, that is, along ⟨11–2⟩ rather than along ⟨110⟩ on the ripple’s facets. This situation is sketched in Figure 7 and explains the rationale for two strongly preferred azimuth directions for the emerged Ge ripples with (√3 × √3)-reconstructed {111}- and {11–1}-oriented side facets. This biaxial local morphology is attributed to a strong stabilization of favorable (111) terraces by the Pt-induced (√3 × √3) reconstruction of the facets. The red circles identify Pt atoms which have replaced Ge atoms in the surface layer. We suggest that this feature even drives the distribution of atomic steps on the (111) facets in favor of a fit of the building blocks to individual terraces giving rise to the so-called magic terraces.^28^ For ease of survey, Figure 7 is organized differently when compared to Figure 5: Still, we show a (110)-oriented projection, but instead of the two sublattices, we only show one here. Deeper lying but still exposed lattice sites are shown with increasingly darker contrast. We only show the {111}-oriented facet, and the equivalent (mirrored) {11–1} facet on the opposite side of the ripple is not shown. The blue lines indicate multisteps on the {111} facet. The red lines on the left-hand terrace indicate three √3 building blocks, while the larger red parallelogram on the central terrace illustrates an entire (3 × 3) unit cell. Note the distortion due to the projection onto {110}. The shown stepped (111) surface has a (7, 3, 5) nomenclature and intersects the macroscopic {110} surface along [−554], that is, the green line in Figure 7. We note that the [−110] line signifies mirror symmetry, and therefore on {110} similar ripples are expected to align along the [−55–4] azimuth. The angle between both azimuth directions amounts to 59°, in agreement with the data displayed in Figure 3d. We consider this finding as strong supporting evidence for the proposed model. From the lack of a peak just in the center between the two major peaks, we can safely conclude that an alignment of the ripples along [−110] is insignificant, which is in agreement with the earlier suggested importance of alignment of Pt-rich chains with steps. We do note that a vertical shift of the domains in Figure 7 along [001] cannot be excluded. In the ideal case, such a shift would result in an angular spacing of 31.6° instead of 59° (cf. thin blank arrows in Figure 3d). The data displayed in Figure 3d show evidence in support of 31.6° next to ∼60°. In addition, a variation of the atomic step distance, for instance, by one atomic building block, would only result in an increase of the angular separation of the ripple orientations by 3.4°, that is, from 59.0 to 62.4°. As an intermediate result, we conclude that the situation sketched in Figure 7 nicely covers the observations.

**Figure 8:**
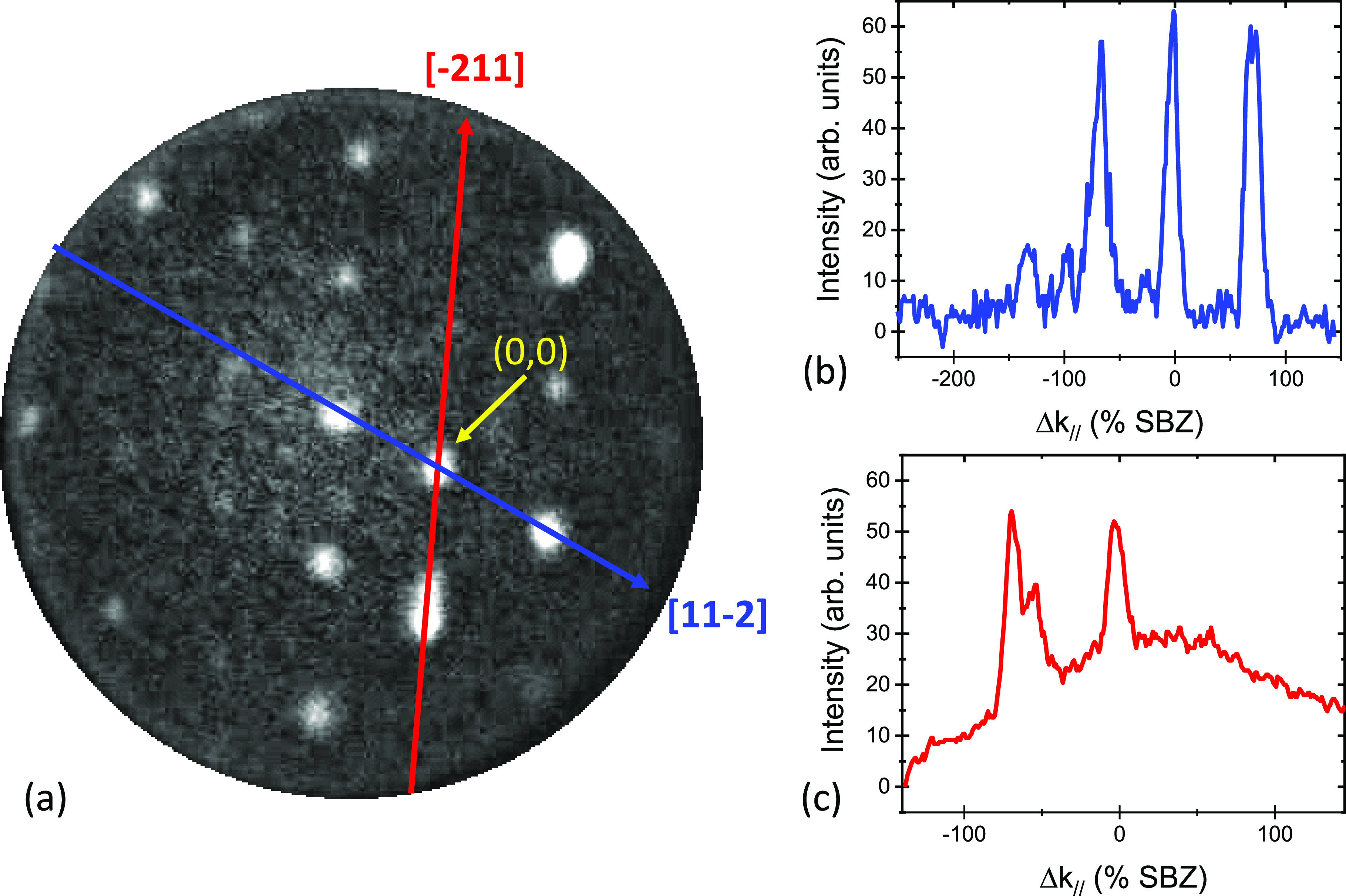
(a) (3 × 3) μLEED pattern obtained at 5.4 eV. The azimuth directions indicate real space directions that correspond to the azimuth direction on {111} with the highest Pt density cf. Figure 7. The intensities have been corrected for the intensity variations resulting from the secondary electron plume. Also indicated by an arrow is what we believe to be the specular spot (0, 0). (b,c) Intensity profiles along [11–2] and [−211].

To gain additional information on the step density along [−554] (cf. Figure 7), we have a closer look at the width of the peaks along [11–2] (real space indication), as displayed in Figure 8. The corresponding intensity profile is shown in Figure 8b. For comparison, we use the intensity profile along [−211] in Figure 8c. Note that both directions correspond to azimuth directions on the {111} facets with the highest Pt density, according to Figure 7. The FHWM of the specular beam along [11–2] (first intense spot from left in b) is about equal to that along [−211] (first intense spot from right in c). After correction for distortion caused by the {110} projection (see also Figures 4 and 6), an additional broadening by a factor of 1.3 along [11–2] remains. As the broadening of the peaks is related to the step density, as described in a detailed fashion in refs (21) and (22), this directly implies that the step density along [11–2] is high. This is in agreement with the discussion on the terrace width in Figure 7 and supports the presence of a significant distribution of step widths along [−554].

**Figure 9:**
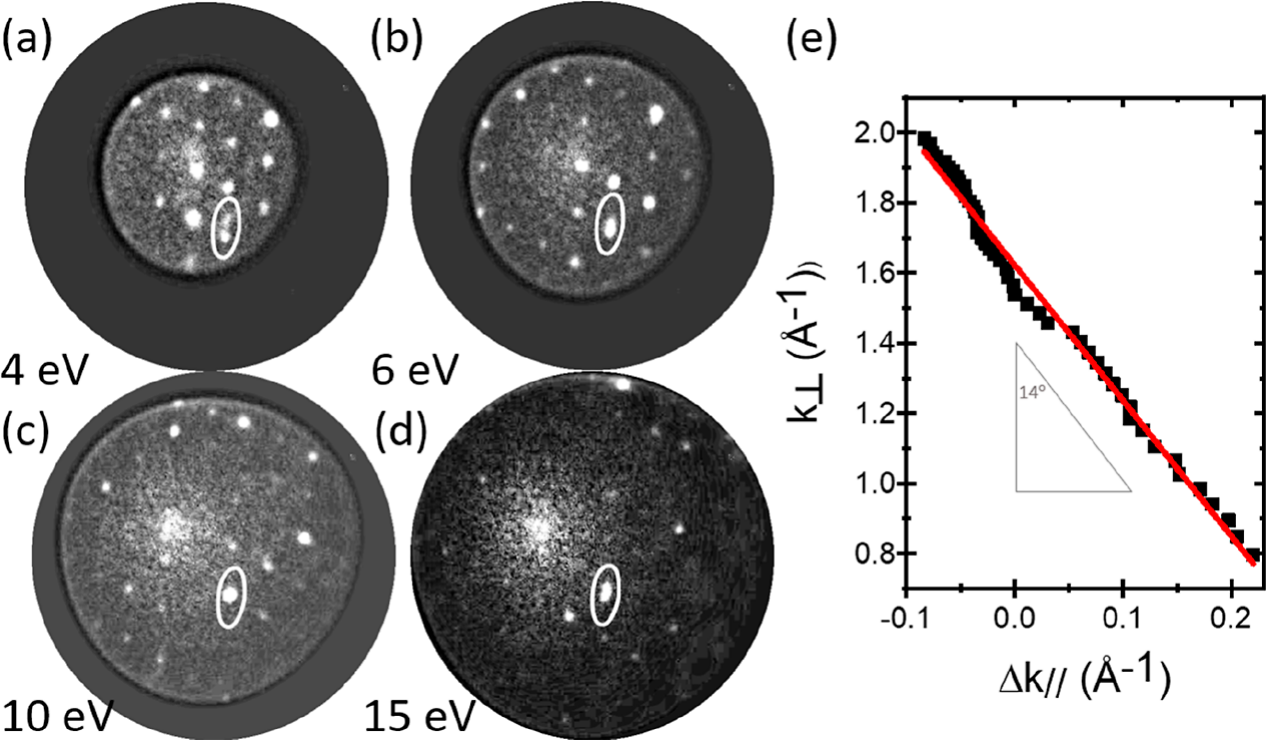
(a–d) μLEED patterns obtained at 4, 6, 10, and 15 eV. Once more, the circles represent the Ewald sphere which scales with *E*^1/2^. The patterns have been corrected for intensity variations resulting from the secondary electron plume. The ellipses illustrate peak splitting along [−211] due to steps at regular distances. The magnitude of this splitting is shown in (e) as a combination of Δ*k*_⊥_ and Δ*k*_//_ obtained at each energy.

Encouraged by this result, we now have a closer look at the step density along “[001]”. For this purpose, we inspect the μLEED pattern obtained for energies between 1.9 and 17 eV. In contrast to the broadening along [11–2] discussed above, we now obtain unambiguous evidence for the presence of well-defined split peak pairs for each energy. Characteristic data obtained at 4, 6, 10, and 15 eV are shown in Figure 9a–d, respectively. Again, this evidence reveals the presence of atomic steps, now at a well-defined distance. According to, once more, Horn von Hoegen,^21,22^ a plot of the changes in the vertical component of the wave vector versus the parallel one directly yields the angle between the stepped facets and the constituting {111} terraces. The result is plotted in Figure 9e for the split pairs measured in the energy window between 1.9 and 17 eV. The obtained angle is about 14°, implying that the separation between [−554] steps is consistent with the presence of about 4√3 building blocks along the direction [−110], cf. Figure 7. Therefore, the slope of the facets of the ripples on each side are about 21° from the [110] surface. The intermediate result for the emergence of the “spaghetti” pattern can be summarized as follows. It consists of ripples which are oriented along, mainly, [−554] and equivalent directions on the {110} surface. The constituting material is Ge released by the spinodal decomposition of the eutectic droplets upon passing the eutectic temperature of the GePt system. The facets of the parallel ripples, (111) vicinals, topped by a Pt-containing layer in a (3 × 3) (or, equivalently, (√3 × √3) R30°) structure in which one-third of the Ge atoms in one of the bilayers, probably the lower one, is replaced by Pt atoms. These facets make an angle of about 21° with {110}.

Above, we have discussed in considerable detail the breakaway of Ge from a huge eutectic GePt droplet during a cool-down through the critical temperature. As we have shown, complex and large-scale pattern formation occurs, leading to ripples with vicinal (111) facets of pure Ge covered by a (√3 × √3) R30° Pt-containing cover layer. The ripples are oriented along, mainly, [−554], [−55–4], and along [−552] and [−55–2] azimuth directions on the {110} surface. Nothing yet has been said about the counterpart, that is, the emergence of Ge_2_Pt crystallites upon passing through the eutectic temperature, in accordance with the phase diagram in Figure 2. The emergence of such crystallites has been reported recently,^16^ and their Ge_2_Pt composition and crystalline structure were established beyond any doubt. Several crystalline shapes were detected, including the so-called elongated hut clusters. These hut clusters have a {001} top face aligned parallel to Ge{110}. An almost perfect match is detected along the [001] azimuth, and twice the periodicity along the [100] azimuth on Ge_2_Pt{100} equals well three times the periodicity along [−110] on Ge{110}. (110) facets complete the hut clusters.

**Figure 10:**
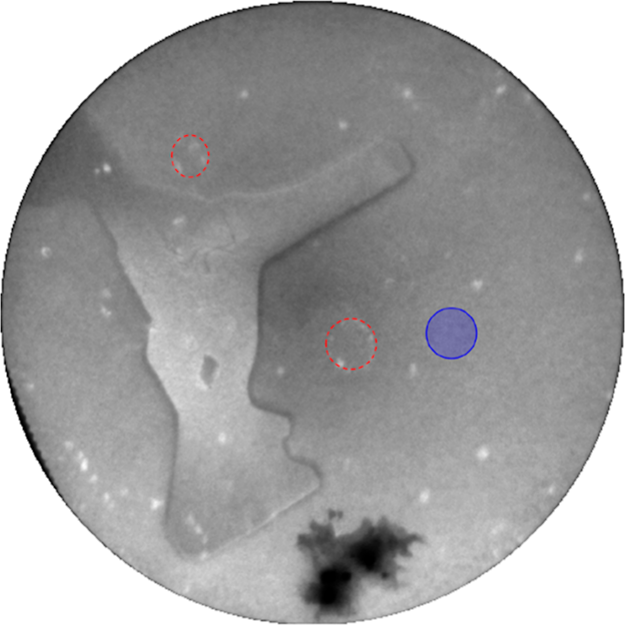
Snapshot from a movie taken at room temperature; FoV, 20 μm. Electron energy, 3.1 eV. The “fairy circles” (dashed red features) are leftovers from the former eutectic droplets originating from previous experiments. The small white features are probably 3D Pt–Ge objects. The irregularly shaped feature near the center is a 2D crystallite. The blue circle is a possible fictive parent droplet of the crystallite with a wetting angle of 20°.

As noted above, both the composition and the overall crystal structure of the emerging Ge_2_Pt are known. However, it appears that the intimate connection of these crystallites with the host substrate depends on the cooling rate. We have conducted μLEED measurements on coagulated clusters at about 1 mm from the center of the surface in a further attempt to unravel the crystalline structure. Only limited coalescence has occurred at these positions, and the passing small(er) clusters are still moving under the influence of the prevailing thermal gradient. As a result, we cannot catch live the ultimate moment of solidification upon passing the eutectic temperature, but we can identify several crystallites after the action. A representative example is shown in Figure 10 obtained at room temperature with 3.1 eV electrons. The small white blobs are probably crystalline Ge–Pt objects but are too small to characterize in μLEED.

**Figure 11:**
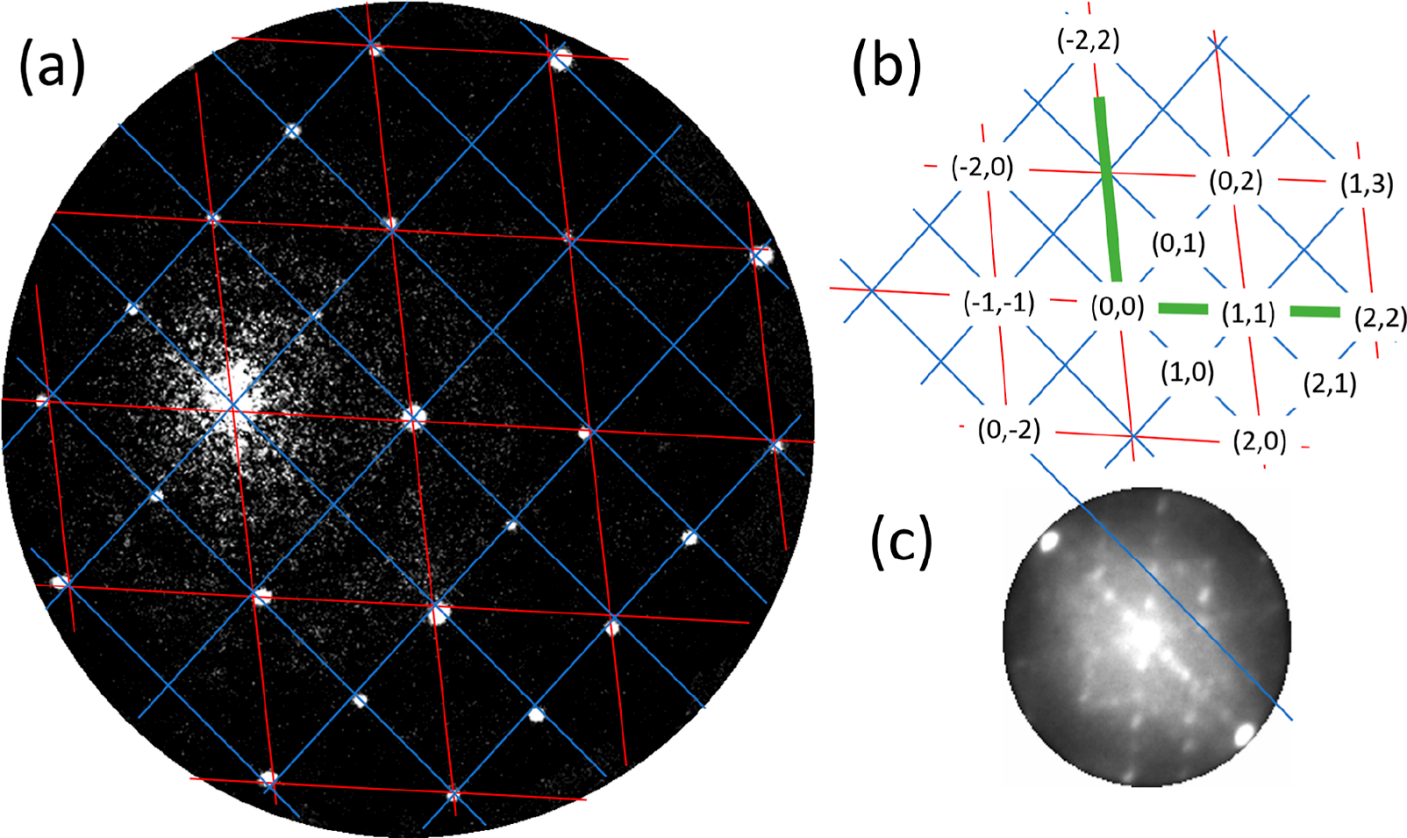
(a) Measured μLEED pattern (44 eV) on top of the 2D Ge_2_Pt island shown in Figure 10. (b) Constructed LEED pattern for a base cell with two Pt atoms on a Ge_2_Pt{101} plane. (c) Diffraction pattern (5 eV) of the Ge{110} substrate next to the 2D Ge_2_Pt island. The blue line indicates the [001] direction.

Sometimes, they are arranged in circular patterns, and these “fairy circles” (see red ellipses) are leftovers of former droplets formed in earlier experiments. The irregularly shaped large feature turns out to be a 2D Ge_2_Pt crystallite, as shown below. The crystalline structure of PtGe_2_ is orthorhombic with *a* = 6.179 Å, *b* = 5.779 Å, *c* = 2.914 Å, and α = β = γ = 90°.^29^ Note that *b* equals the Ge lattice constant within about 2%, and commensurability is thus obtained along Ge[001] if the contact plane is along the Ge_2_Pt{101} plane, with the *b*-axis parallel to Ge[001]. The μLEED diffraction pattern obtained on top of the Ge_2_Pt island in Figure 10 is shown in Figure 11a, together with the expected diffraction pattern in Figure 11b a and the diffraction pattern of the Ge{110} substrate next to the Ge_2_Pt island in Figure 11c. The blue rectangular grid in reciprocal space is indeed aligned along the Ge[001] direction and corresponds to the centered two-atomic base expected for the unit cell considering the Pt atoms only. The red grid shows the primitive lattice for the Ge_2_Pt{101} contact plane. The blue lattice is slightly distorted when compared to the theoretical rectangular version (compare the top view in Figure 13a. Given this limitation, a convincing agreement is obtained between the measured and the constructed patterns. Note that the ratio between the sides of the found rectangle equals within a few percent the theoretically expected one.

**Figure 12:**
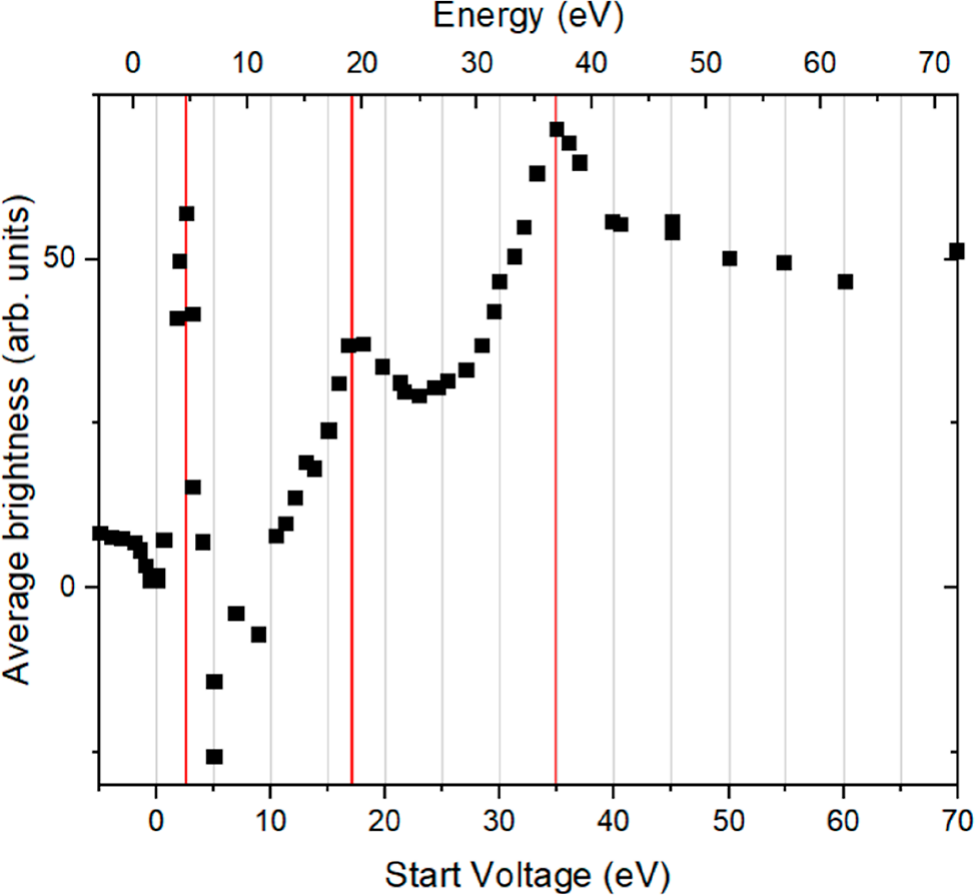
Average brightness of a representative area on top of the island minus a ditto on the substrate versus the applied start voltage. The energy scale at the top was shifted by 2.0 eV to account for the contact potential between the electron source and the sample and the inner potential.

**Figure 13:**
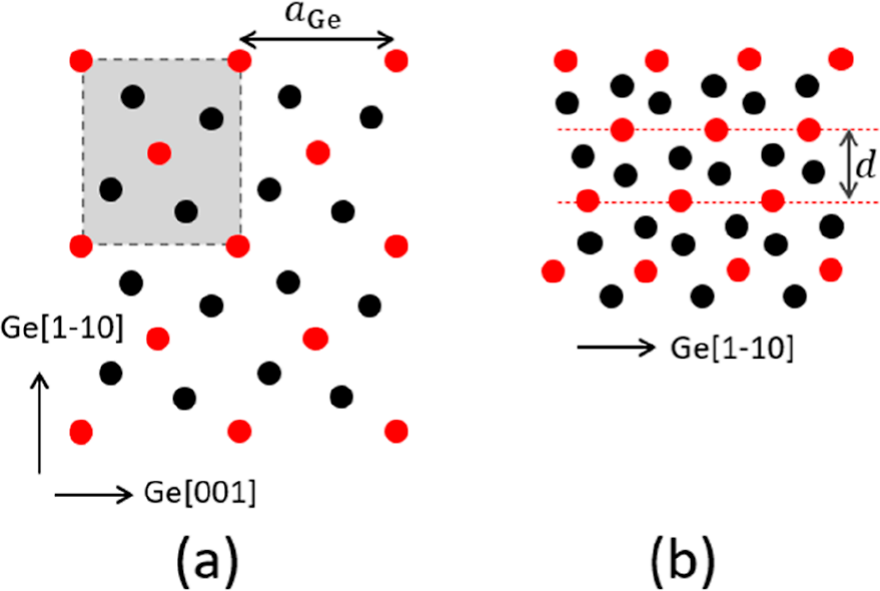
(a) Top view on the Ge_2_Pt{101} crystallite: red points represent Pt atoms, and black points indicate Ge atoms, projected onto the Ge_2_Pt{101} plane. The centered unit cell is highlighted. (b) Side view of the Ge_2_Pt{101} crystallite along the [100] azimuth in Ge{110}. The distance between the Pt planes is *d.*

Information on the vertical parameters of the crystallite is accessible by an intensity analysis of the intensity versus electron energy data obtained in μLEED on top of the Ge_2_Pt island. To correct for the ,influences of the electronic structure of the surface we have subtracted the intensity from a representative area of the substrate. Such a difference curve is shown in Figure 12. Clear features are observed in the low-energy range, with three very prominent features at about 3, 17, and 35 eV. Oscillatory intensity variations in electron scattering from thin objects are commonly attributed to the Fabry–Pérot behavior, due to the interference between scattering contributions from the surface and the hidden interface.^30^ On the square root of the energy scale, the intensity maxima would appear at equidistant separations. The number of maxima up to the Bragg peak would equal the number of layers minus 1.

**Figure 14:**
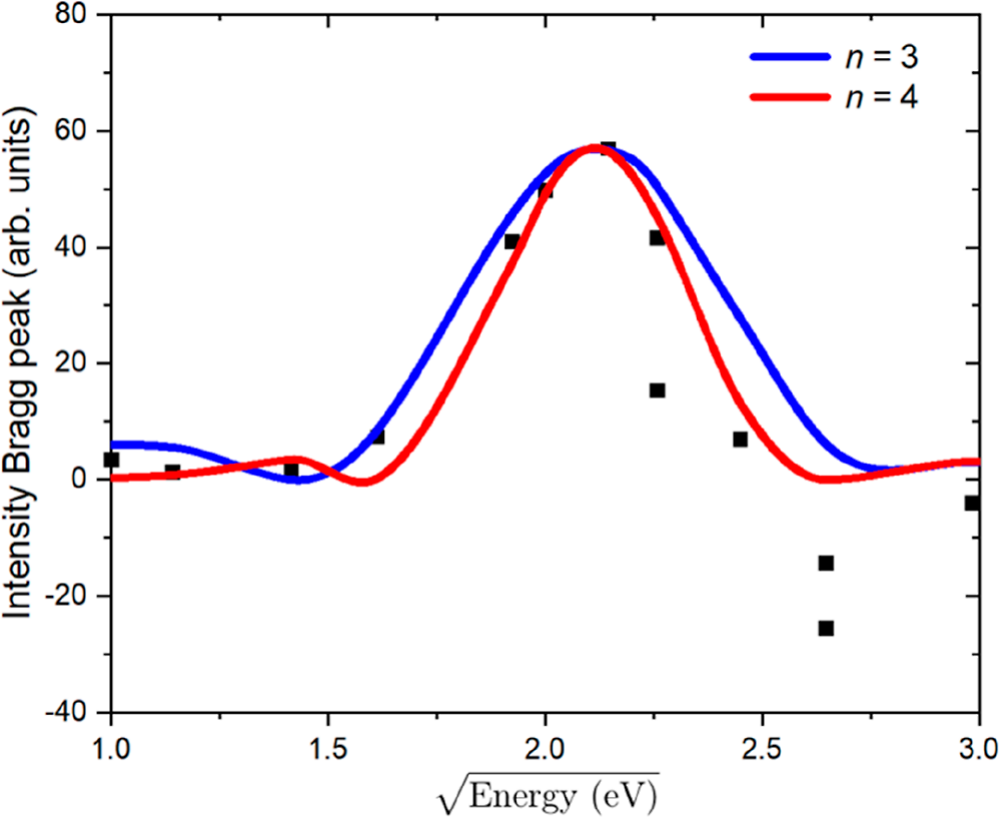
Measured first-order Bragg peaks (black squares, from Figure 12) and calculated profiles using eq 1 for *n* = 3 (blue) and for *n* = 4 (red).

The fit in Figure 12, as indicated by the red lines, seems in line with this interpretation. The thin film would then be three layers thick; the contact potential between the electron source and the Ge_2_Pt{101} film amounts to a realistic 1.7 eV. A severe problem emerges because the Bragg peak resides at 37.5 eV, which would indicate an interlayer distance of only 1 Å. For comparison, we show in Figure 13b a side view of the thin Ge_2_Pt{101} film along its *b*-axis. This [001] direction is oriented along the normal to the plane of drawing. The Pt atoms are shown as red circles and the Ge ones as black ones. The distance between the Pt planes along the normal to the contact plane amounts to 2.64 Å. The Bragg condition for specular diffraction from this structure is then given by λ′ = 5.28 Å, with λ′ being the internal wavelength of the probing electrons. This anticipated interplanar distance of 2.64 Å is in clear contrast with the obtained value of 1.0 Å, and we thus must search for another solution. This is found straightforwardly by attributing the left-hand red peak in Figure 12 to the Bragg peak of the film structure. By assuming in all respects a realistic value of 1.7 eV for the inner potential in the Ge_2_Pt{101} film, one arrives at the expected interplanar distance of 2.64 Å. The two remaining peaks in Figure 12 are then directly assigned as the second and third order Bragg peaks. Unfortunately, we seem to lose the option to obtain information on the thickness of the film as we do not observe maxima at the left-hand side of the Bragg peak in Figure 12. The close vicinity to mirror imaging excludes this possibility, as well as the increasingly poorer definition of the wavelength at these low energies. Fortunately, we have access to the thickness of the film by the examination of the width of the (first) Bragg peak. For a slab consisting of *n* layers at a mutual interplanar distance *d*, the shape of the Bragg peak is given by
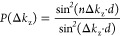
1with Δ*k*_z_ = π*/*λ, and λ is the electron wavelength. The calculated profiles P(Δ*k*_z_) for *n* = 3 and *n* = 4 are plotted in blue and red, respectively, in Figure 14, where they can be compared to the experimental result (black squares), replotted from Figure 12. The best result is obtained for *n* = 4. From this, we derive that the Ge_2_Pt{101} island from Figure 11 has an estimated thickness of four layers. Hence, it appears that micron-sized droplets solidify in extended flat islands. A natural reason for this preferred thickness is found in quantum size stabilization. This occurs under the condition^31^
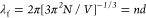
2where λ_*f*_ is the Fermi wavelength, *N*/*V* is the free electron density in Ge_2_Pt, *n* is an integer number, and *d* is the interlayer distance in the Ge_2_Pt{101} film. The ionization energy for Pt in a Ge surrounding is 40 meV.^32^ At 1050 K, the degree of ionization then amounts to 0.38, and the Fermi wavelength, λ_f_, equals 10.4 Å. From eq 2, we derive that *n* = 4.0, that is, the thin film is four layers thick, in agreement with the data in Figure 14. We conclude that the stabilization of the observed thin Ge_2_Pt{101} film by quantum size effects is a plausible cause for the development of extended thin Ge_2_Pt{101} crystallites upon the solidification of micron-sized droplets when passing the eutectic temperature. With the known wetting angle of 20° before solidification,^13^ one can estimate the projected view of a fictive spherical cap as the parent droplet for the flat island in Figure 10. The result is given by the blue object in Figure 10. Its size compares well with the fairy circles indicated by the dashed red features as the presumed remnants of disappeared droplets in previous experiments.

**Figure 15:**
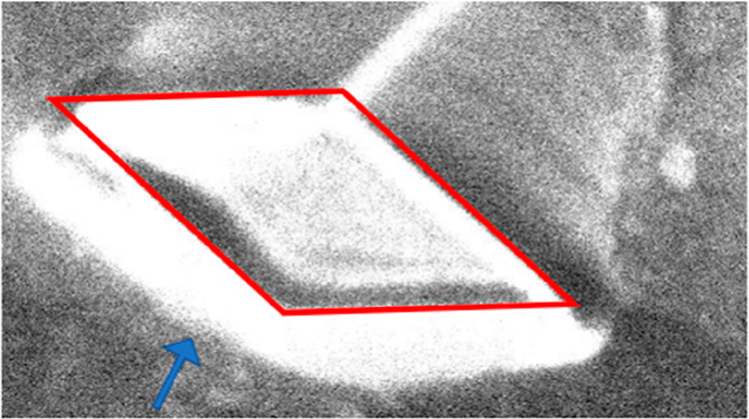
PEEM image at the substrate center of a huge Ge_2_Pt rhombic crystallite after solidification during cooling through the eutectic temperature; FoV, 80 × 45 μm. Its location is in the center, where, before cooling, a huge droplet had evolved. The UV light is incident along the blue arrow. The edges of the rhombus are emphasized by the black lines. See text for further details.

**Figure 16:**
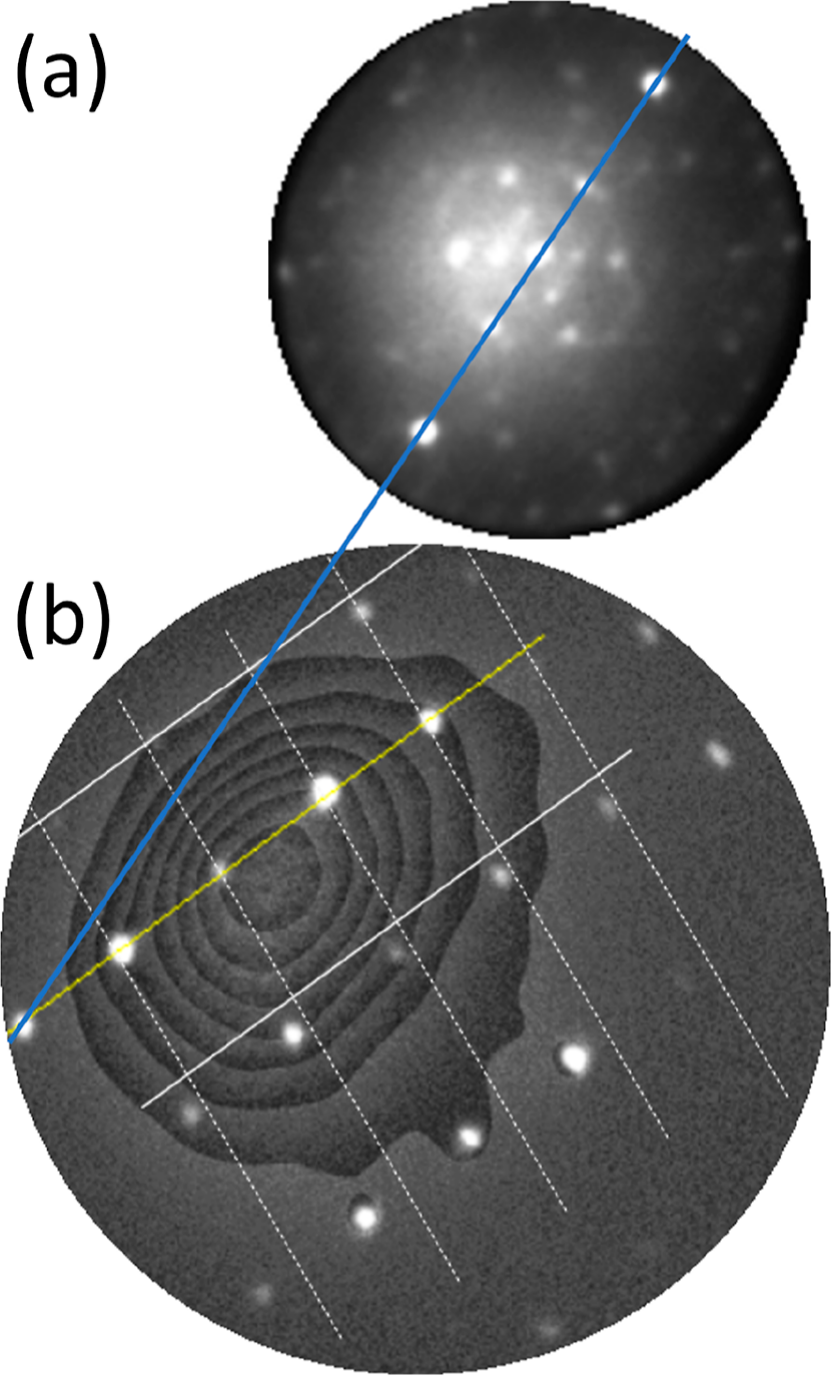
(a) μLEED pattern (8 eV) of the Ge(110) surface underlying the huge rhombus. The blue line indicates the (001) direction. (b) μLEED pattern taken at 25 eV on top of the huge rhombus (see Figure 15). The white grid shows a reciprocal lattice obtained by assuming a {001}-oriented Ge_2_Pt structure on top of the Ge{110} structure (see text). A huge distortion is seen mainly perpendicular to the yellow line. The grid even looks aperiodic.

**Figure 17:**
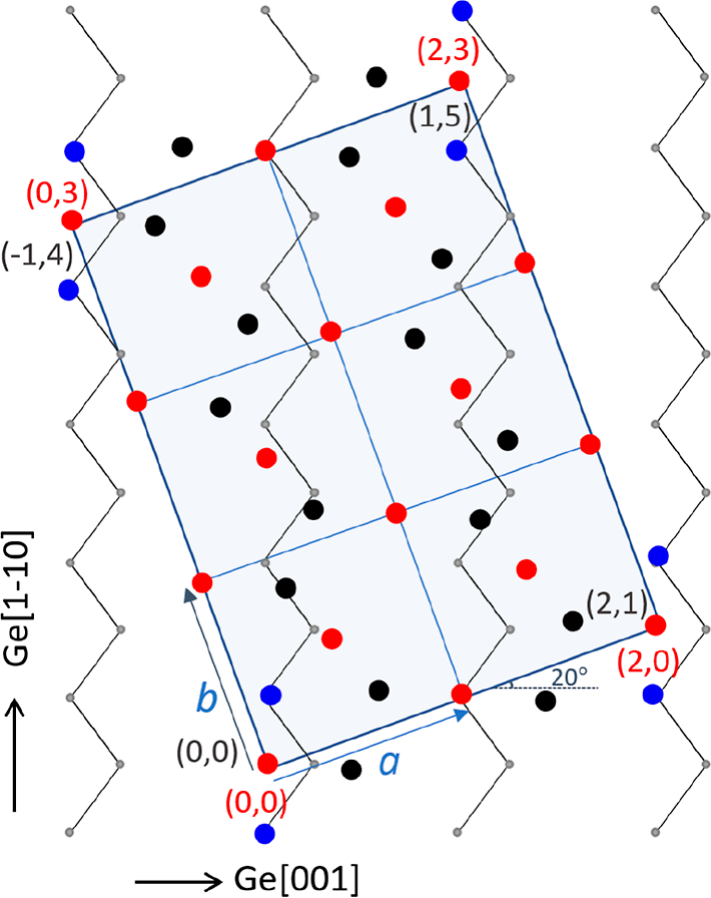
Top view of Ge_2_Pt{001}–Ge{110} contact plane. The gray zig-zag lines represent the Ge top layer (with the Ge atoms positioned at the kinks). A translation of the zig-zag lines by half the unit cell diagonal would show the position of the Ge atoms in the second layer at a depth of 2 Å, which is omitted here for simplicity. The orientation of the *a*- and *b*-axes of the Ge_2_Pt crystallite is indicated by arrows. The interlayer distance for bulk Ge_2_Pt{001} amounts to 1.5 Å. The horizontal runs parallel to the Ge[001] azimuth. The light blue rectangle highlights the unit cell, composed of six Ge_2_Pt{001} unit cells. The indices in gray refer to Ge{110} and those in red denote the Ge_2_Pt{001} grid. Note that only the Pt (red) and Ge atoms (black) in the contact plane are shown. In addition, the sites at the corners of the unit cell form a tight connection to the substrate, as Ge positions in both crystals are common here. These positions are indicated by the blue Ge atoms.

So far, we concentrated on the solidification of micron-sized clusters at ca. 1 mm away from the center of the substrate. Encouraged by the detailed information on the solidification during cooling and on the emerging crystalline structure of the Ge_2_Pt clusters we were able to unveil, we now concentrate on the solidification of the single huge cluster that evolved near the center of the substrate during cooling through the eutectic temperature.^13^ The resulting compact rhombic Ge_2_Pt crystallite is shown in the PEEM image in Figure 15. The azimuth of the illuminating UV light is indicated by the blue arrow.^12,13^ The red rhombus accentuates the edges of the rhombus (see further below for more details). The crystalline rhombus is a huge monolith on the atomic scale with a long axis of ca. 75 μm. Its height is estimated at several tens of microns too. We have recorded μLEED patterns on top of the rhombus in spite of substantial distortions due to huge field inhomogeneities^33^ around this tall object. The result is shown in Figure 16. Apart from a sizeable distortion about perpendicular to the yellow line, a decent diffraction pattern emerges, which is reasonably well represented by the white rectangular white grid. Based on the comparison with the Ewald circle at 25 eV, we estimate the length of the sides of the rectangle at about 6 Å. This would correspond to a Ge_2_Pt{001} unit cell. The pattern deviates markedly from the one obtained for thin extended crystallites evolving from smaller droplets located at 1 mm or more from the center (cf. Figure 11). This is best illustrated by the about 20° different azimuthal alignment of the grid with respect to that of the substrate (see Figure 16b). Consequently, we suggest that the compact tall cluster constitutes a {001}-oriented Ge_2_Pt film, with its major axes rotated away from the [001] azimuth on Ge{110}. A clue for the 20°-rotated azimuth orientation, when compared to the aligned flakelets in Figure 10, is revealed by inspecting the interface in more detail. A representative sketch of the contact plane is shown in Figure 17. The top layer of the Ge{110} substrate is shown by gray atoms, situated at the kinks in the gray zig-zag rows. The horizontal equals the [001] substrate direction. The bottom layer of the Ge_2_Pt{001} crystallite is indicated by red and black circles for the Pt and Geatoms, respectively. The grid is rotated by 20° (*a*-axis wrt the Ge{110}–[001] direction), and along the *a*-axis, an almost perfect match is obtained for every second Ge_2_Pt trimer (mismatch 2%). This provides solid anchor sites for the crystallite on the substrate, as Ge positions in both crystals are common here (indicated by the blue atoms in Figure 17). Along the *b*-axis, every third atom string offers similarly an excellent match. We consider these findings as strong evidence for the unanticipated azimuthal relationship for Ge{110} and Ge_2_Pt{001}, with an angle of 20° between the *a*-axis and the Pt[001} axis. The anticipated superstructure for the substrate is denoted by  and coincides with the (2 × 3) Ge_2_Pt{001} mesh (seeFigure 17). We suggest that first the stable strings consisting of a series of linear Ge2Pt trimers, aligned along the [1–12] direction (cf. Figure 17), may form. With increasing density, the strings are pushed together to build the first Ge_2_Pt{001} layer as a base for the tall crystallite.

The preference for the *a*-axis at 20° from Ge[001] is expected to show in the shape of the tall compact Ge_2_Pt{001} crystallite. For this purpose, we have indicated the directions at ±20° in Figure 15 (see the 2D rhombus with red sides). Note that we have no way of indicating the exact Ge[001] direction in this figure but have to conclude that it is close to perpendicular to the blue arrow in Figure 15. The strong preference for edges at angles of about 40° is evident.

Summarizing the final part, we conclude that the spinodal decomposition of eutectic Ge–Pt droplets leads to the evolution of Ge_2_Pt crystallites. For smaller droplets located at 1 mm or more from the center, we find Ge_2_Pt{101}-oriented films, with the *b*-axis aligned parallel to Ge[−110]. These films remain ultrathin with a thickness of about four layers and are stabilized by quantum size effects. The spinodal decomposition of the huge droplet in the center of the substrate leads to the formation of a large, compact Ge_2_Pt{001} crystallite, with again the *a*-axis pinned at 20° from Ge[−110].

## Conclusions

In summary, we have studied the spinodal decomposition of PtGe droplets *in situ* using LEEM, PEEM, and μLEED. During the initial fast cooling toward the eutectic temperature, the droplet’s footprint shrinks due to the segregation of Ge from the liquid into the Ge substrate, accompanied simultaneously by an increase of the solid–liquid interface tension due to kinetic amorphization at the droplet–substrate interface. During the subsequently lower cooling rate, the amorphous crystalline interface recrystallizes, accompanied by a decrease of the liquid–substrate interface tension and an increase of the droplet’s footprint.

Upon passing the eutectic temperature, spinodal decomposition occurs, resulting in PtGe_2_ crystallites accompanied by a spreading of the excess Ge into a complex pattern of ripples with vicinal (111) facets of pure Ge covered by a (3 × 3) Pt-containing superstructure. The ripples are oriented along, mainly, [−554], [−55–4], and along [−552] and [−55–2] azimuth directions on the {110} surface. Spinodal decomposition of the large droplet in the center resulted in a compact rhombic Ge_2_Pt crystallite with a {001}-oriented Ge_2_Pt–Ge(110) interface, with the *a*-axis pinned at 20° from Ge[−110].

For smaller droplets located at 1 mm or more from the center, and thus lower local Pt content, we find Ge_2_Pt{101}-oriented films with the *b*-axis aligned parallel to Ge[−110]. These films remain ultrathin with a thickness of about four layers and are stabilized by quantum size effects.

## Supporting Information Available

PEEM movie of the eutectic PtGe droplet at the hot spot of the sample during cooling toward the eutectic temperature, accompanied by a live graph of the temperature and the projected area of the large eutectic droplet, normalized to its initial value as a function of time (MP4)
